# Supplementary material for: Emergency SARS-CoV-2 Variants of Concern: Novel Multiplex Real-Time RT-PCR Assay for Rapid Detection and Surveillance
Source: Microbiol Spectr. 2022 Feb 23;10(1):e02513-21. doi: 10.1128/spectrum.02513-21 (PMC8865422; doi:10.1128/spectrum.02513-21)
Supplement: SUPPLEMENTAL FILE 1 — Supplemental material. Download SPECTRUM02513-21_Supp_1_seq12.pdf, PDF file, 0.2 MB [file spectrum02513-21_supp_1_seq12.pdf]

Supplementary Table 1. Analytical sensitivity of the RT-qPCR VOC assay for SARS-CoV-2 testing.

Target gene	Control Concentration (copies/μL)	LightCycler® 96 (Ct value)		LabTurbo AIO (Ct value)	
		Mean	95% CI	Mean	95% CI
N501Y	60	30.11	29.96-30.25	30.14	30.01-30.27
	30	31.83	31.49-32.17	31.87	31.79-31.95
	15	32.95	32.37-33.53	33.02	32.37-33.66
Del 69-70	60	30.47	30.2-30.73	30.68	30.38-30.97
	30	31.90	31.51-32.29	31.63	31.27-31.99
	15	32.90	32.49-33.29	32.41	32.00-32.83
E484K	60	30.66	30.43-30.89	30.11	29.97-30.25
	30	31.51	31.27-31.75	31.78	31.56-32.01
	15	33.14	32.86-33.43	33.23	32.91-33.55

K417N	60	30.61	30.33-30.88	30.50	30.26-30.72
	30	31.84	31.34-32.34	31.76	31.33-32.20
	15	32.87	32.47-33.28	32.78	32.36-33.20
L452R	60	30.21	29.89-30.52	30.17	29.90-30.43
	30	31.56	31.31-31.81	31.39	31.10-31.69
	15	33.26	32.91-33.62	33.18	32.89-33.46
P681R	60	30.59	30.38-30.81	30.69	30.45-30.85
	30	31.16	30.97-31.30	31.62	31.33-31.84
	15	33.21	32.98-33.52	33.61	33.17-33.61

Supplementary Table 2. VOC typing results compared with Whole genome sequencing in Omicron variant

		VOC RT-PCR		Whole genome sequencing	
		Omicron (B.1.1.529)		Omicron (B.1.1.529)	
Spike region	Spike variation	BA.1 (n=5)	BA.2 (Stealth version) (n=5)	BA.1 (n=5)	BA.2 (Stealth version) (n=5)
HV69/70	Δ HV69/70	-	+	+	-
K417	K417N	+	+	+	+
N501	N501Y	+	+	+	+
P681	P681R	+	+	+	+
